# Anaesthetic Management of Foreign Body Bronchus at University Teaching Hospital of Nepal: An Observational Study

**DOI:** 10.31729/jnma.9012

**Published:** 2025-05-31

**Authors:** Megha Koirala, Bashu Dev Parajuli, Pankaj Joshi, Basanta Ghimire, Amit Sharma Bhattarai

**Affiliations:** 1 Department of Anesthesiology, Tribhuvan University Teaching Hospital, Maharajgunj Medical Campus, Institute of Medicine, Maharajgunj; 2 Department of Cardiothoracic Vascular Anesthesia, Manmohan Cardiothoracic Vacular Centre, Maharajgunj Medical Campus, Institute of Medicine, Maharajgunj

**Keywords:** *foreign body bronchus*, *general anesthesia*, *rigid bronchoscopy*

## Abstract

**Introduction:**

Rigid bronchoscopy is the preferred method for extracting airway foreign bodies, as it allows grasping forceps to extract foreign bodies while maintaining ventilation through a side port. The main challenge to the Anesthesiologists is to maintain oxygenation and ventilation while sharing the common field with the surgeons. This study intends to evaluate our current practices, challenges, and intraoperative events to enhance patient safety during these procedures.

**Methods:**

This is a prospective observational study that included patients with suspected or confirmed bronchial foreign bodies who underwent rigid bronchoscopy from February 2023 to February 2025. Data were collected by the anesthesiologists using the structured proforma, covering the anesthetic technique, complications, and details of foreign bodies. SPSS version 22 was used for analysis.

**Results:**

A total of 41 rigid bronchoscopies were performed, including three repeat procedures. Intraoperative complications included desaturation in 29 (70.73%), vocal cord edema in 16 (39.02%), slipping of foreign body in 8 (19.51%), and both bradycardia and airway bleeding in 6 (14.63%) cases each. Postoperatively, 23 (56.10%) patients required mechanical ventilation, and one (2.44%) patient experienced cardiac arrest. During preoperative preparation, intravenous glycopyrrolate and steroids were used in 38 (92.68%) and 34 (82.93%) cases, respectively. For induction and maintenance, intravenous anesthesia was the most common technique, with muscle relaxants used in 39 (95.12%) cases. Of the 41 bronchoscopies, 30 (73.17%) achieved successful foreign body removal, while five (12.20%) required thoracotomy referrals.

**Conclusions:**

Desaturation was the most common intraoperative complication. About half of the patient required mechanical ventilator postoperatively.

## INTRODUCTION

Rigid bronchoscopy is the preferred technique for removing airway foreign bodies, as it allows the use of grasping forceps to extract the object while simultaneously providing ventilation through a side port.^1^ These cases present several anesthetic challenges. A major concern is the risk of complete airway obstruction, which may cause oxygenation deprivation, potentially resulting in hypoxia and, if not addressed, cardiac arrest.^2-6^ Vigilance and effective communication with the surgical team are essential to mitigate such risks. At our institute, rigid bronchoscopy is usually performed under general anesthesia with muscle relaxation, using the side port for ventilation. Inhalational agents or total intravenous anesthetics are used for maintenance. Patients are typically extubated after foreign body removal if no adverse events occur. This study assesses our current practice, anesthetic challenges, and intraoperative events during rigid bronchoscopy for foreign body removal.

## METHODS

This is an observational study conducted at Tribhuvan University Teaching Hospital (TUTH) which is a tertiary care university hospital situated at Kathmandu, Nepal. This study included all patients, either suspected or confirmed to have a foreign body in the bronchus, who underwent rigid bronchoscopy over two years (February, 2023-2025). The study was conducted following approval from the Institutional Review Committee [Reference number: 397(6-11)E2]. Data collection was carried out using a structured proforma by an anesthesiologist responsible for obtaining the patient's history and managing anesthesia during the procedure. The proforma was completed on the same day after completing the procedure. Key recorded information included the anesthetic technique used for airway management, any complications encountered, pre-procedure preparations, challenges in foreign body retrieval, its migration, the need for post-procedure mechanical ventilation, and referrals for thoracotomy. After the procedure, the primary Ear Nose and Throat (ENT) surgeon verified the collected data. Subsequently, another investigator entered the data into an Excel sheet, which was later transferred to IBM SPSS Statistics for Windows, version 22 (IBM Corp., Armonk, N.Y., USA) for analysis. Descriptive statistics were applied to summarize frequencies.

## RESULTS

During the study period, a total of 41 rigid bronchoscopies were performed, including three repeat procedures due to unsuccessful foreign body removal on the initial attempt. Consequently, the total number of individual patients analyzed for age and sex was 38, while each bronchoscopy was considered a separate case for all other variables. A total of 18 (47.37%) patients were in the 1-5-year age range, with a male-to-female ratio of 2.4:1. Only 11 (28.95%) cases sought medical advice within the first 24 hours, while 27 (71.05%) presented after a delay. Fourteen (36.84%) patients required oxygen upon arrival for bronchoscopy, including one (2.63%) who had been intubated following a failed initial removal attempt. The details of the patients are provided in (Table 1).

**Table 1:** Specification of patients undergoing management of foreign body in bronchus (n=38).

S.	No Patient's Specification	n(%)
1	Age (n=38)	5 (13.16)
	<1 year	5 (13.16)
	1yr-5 years	18 (47.37)
	5-15 years	10 (26.32)
	>15 years	5 (13.16)
2	Sex (n=38)	
	Male	27 (71.05)
	Female	11 (28.95)
3	Time of presentation (n=38)	
	Within 24 hours	11 (28.95)
	24 hours to 7 days	18 (47.37)
	More than 7 days	9 (23.68)
4	Attempt at bronchoscopy (n=41)	
	First	38 (92.68)
	Second	3 (7.32)
5	Oxygen requirement at presentation (n=41)	14 (34.15)

**Table 2:** Anaesthetic management of patients undergoing management of foreign body in bronchus (n=41).

S. No	Anaesthetic specification	n(%)
1	Preoperative preparation	
	Nebulization	12 (29.27)
	Intravenous Glycopyrrolate	38 (92.68)
	Intravenous Steroid	34 (82.93)
	Intravenous Antiboitics	6 (14.63)
2	Modality of ventilation	
	Controlled	39 (95.12)
	Spontaneous	2 (4.88)
3	Induction Technique	
	Inhalation	6 (14.63)
	Intravenous	34 (82.93)
	Mixed	1 (2.44)
4	Maintenance Technique	
	Inhalation	0 (0)
	Intravenous	28 (68.29)
	Mixed	13 (31.71)
5	Use of Muscle relaxant	
	Yes	39 (95.12)
	No	2 (4.88)

**Table 3:** Fate of the foreign body bronchous (n=41).

S. No	Foreign body specification	n(%)
1	Outcome of rigid bronchoscopy	
	No foreign body	6 (14.60)
2	Type of foreign body	
	Organic Seeds	19 (46.30)
	Metallic	3 (7.32)
	Pen back cover	6 (14.63)
	Plastic	2 (4.88)
	Whistle	2 (4.88)
	Wooden block	1 (2.44)
	Chicken bone	1 (2.44)
	Denture cap	1 (2.44)
3	Location	
	Rt. bronchus	17 (41.46)
	Lft. bronchus	10 (24.39)
	Trachea	7 (17.07)
	Subglottic	1 (2.44)
4	Foreign body removal	
	Complete(First setting)	27 (65.85)
	Complete(Second setting)	3 (7.32)
	Impossible	5 (12.20)

Glycopyrrolate was administered to 38 (92.68%) patients, while steroids were given to 34 (82.93%) patients prior to the procedure. Intravenous anesthesia was used for induction in 34 (82.93%) cases and for maintenance in 28 (68.29%) cases. Muscle relaxants were used in 39 (95.12%) cases. Controlled ventilation was employed in 39 (95.12%) bronchoscopies, while spontaneous ventilation was used in 2 (4.88%) cases. The details of anesthetic management are provided in (Table 2).

**Figure 1:**
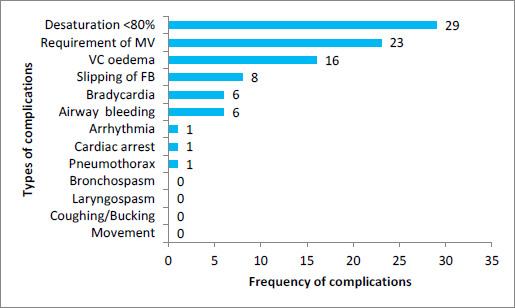
Anaesthetic Complications during Rigid bronchoscopy (n=41).

Intraoperative complications included desaturation (<80%) in 29 (70.73%) cases, while bradycardia and airway bleeding were each observed in 6 (14.63%) cases. Vocal cord edema was observed in 16 (39.02%) cases, while foreign body displacement occurred in 8 (19.51%) cases. Other complications were rare, with arrhythmia occurring in 1 (2.44%) case. Notably, there were no instances of bronchospasm, laryngospasm, movement, or coughing. Postoperatively, 23 (56.10%) patients required mechanical ventilation, and 1 (2.44%) patient experienced cardiac arrest and pneumothorax (Figure 1).

Out of the 41 bronchoscopies conducted, 6 (14.63%) were purely diagnostic, as no foreign body was detected. Organic seeds were the most frequent foreign bodies, found in 19 (46.34%) cases, with 17 (41.46%) lodged in the right bronchus. Each rigid bronchoscopy attempt was counted as a separate case; however, there were actually 38 patients, 3(7.32%) of whom required a second attempt. Successful and complete removal of the foreign body was achieved in 30 (73.17%) cases, while 5 (12.20%) patients required referral for thoracotomy (Table 3).

## DISCUSSION

The most common complication observed during the procedure was desaturation, occurring in 29 cases. Since both surgeons and anesthesiologists work in the same space where a foreign body causes obstruction, desaturation remains the most frequently encountered issue, as reported in previous studies.^3-5^ Desaturation can lead to bradycardia, which was noted in only six cases in our study. The low incidence of bradycardia may be attributed to the use of glycopyrrolate (93%) as a premedication, which helps prevent vagal-induced bradycardia.^6^ Other complications, such as bronchospasm, laryngospasm, coughing, and movement, were rare in our study, likely due to the administration of muscle relaxants and the maintenance of a deep anesthesia throughout the procedure. Additionally, glycopyrrolate may contribute to keeping the airway dry and reducing cholinergic-induced bronchoconstriction.^6^

In our study, the need of postoperative ventilatory support was higher (56%) compared to other study,^7^ likely due to 34% of patients required oxygen upon presentation, and 39% exhibiting vocal cord edema at the end of the procedure. Given these risks, it is crucial to ensure the availability of mechanical ventilator support during such procedures. Steroids were administered preoperatively in 34 cases to reduce airway edema.^2^ Airway bleeding was reported in six cases, while foreign body slippage occurred during eight bronchoscopies.

Anesthesiologists must remain highly alert, vigilant and maintain contant communication with the surgeon throughout the procedure. Positive pressure ventilation should be momentarily paused when the foreign body is being grasped and extraced with forceps. If the foreign body slips during removal, it may result in complete airway obstruction and respiratory failure. In such cases, pushing the foreign body further down may be necessary to ensure the ventilation of atleast one lung. In severe instances of profound hypoxia, surgeons may need to halt the procedure and remove the bronchoscope, allowing anesthesiologist to effectively ventilate the patient.^8^

A critical complication observed in an intubated patient, who experienced cardiac arrest in the post-procedure period after an unsuccessful attempt to remove the foreign body. Cardiopulmonary resuscitation (CPR) was initiated, and return of spontaneous circulation (ROSC) was achieved after three CPR cycles, along with chest tube insertion to manage bilateral pneumothorax. The patient was promptly transferred to the operating room, where the dislodged foreign body was successfully removed from trachea.

Following the procedure, oxygen saturation improved, and the patient was extubated after two days and later discharged without any hypoxic sequelae.

In terms of ventilation, controlled ventilation was utilized in all rigid bronchoscopy procedures except for two cases, where spontaneous respiration was maintained. The preferred ventilation remains a subject of debate.^9^ Some support spontaneous breathing during bronchoscopy to reduce the risk of complete airway obstruction though maintaining it can be challenging. ^8,10^ Striking the right balance in anesthetic depth is crucial—inadequate depth may lead to movement, laryngospasm, and awareness, while excessive depth can suppress respiration.^5^ When muscle relaxants are administered, the anesthesiologist must provide positive pressure ventilation. Although this approach carries a risk of foreign body displacement, it also offers significant advantages. Studies have found no significant difference in the incidence of adverse events between spontaneous and controlled ventilation.^11^ Muscle relaxation helps prevent laryngospasm and involuntary movements, facilitating a smoother and safer foreign body removal.^5^ The use of muscle relaxants and controlled ventilation may attributed the lower incidence of airway trauma, laryngospasm, and patient movement observed in our study. A survey among pediatric anesthesiologists found that nearly half (48.3%) favored controlled ventilation.^12^ Ultimately, the choice of ventilation mode depends on the anesthesiologist's preference. There is no fixed protocol for anesthetic management of airway foreign bodies at our institution, and the high use of muscle relaxants and preference for positive pressure ventilation reflect the choices of the anesthesiologists working here.

In the two cases where spontaneous ventilation was maintained, sevoflurane was used for induction and a mixed technique (inhalation + dexmedetomidine infusion + propofol boluses) was used for maintenance. For the 39 controlled ventilation cases, all patients received propofol either alone or in combination with an inhalational agent (13 cases) or ketamine (6 cases). Some studies suggest that sevoflurane-based induction and maintenance provide superior hemodynamic stability, quicker induction, and faster recovery under spontaneous breathing than intravenous anesthesia.^9^ However, at our institution, anesthesia was primarily induced and maintained using intravenous agents. The preference for intravenous anesthesia in our cases is likely due to concerns of achieving an adequate anesthetic depth when using muscle relaxants. With an open airway, inhalational agents may escape, potentially leading to inadequate anesthesia depth, which could cause patient movement, awareness, and a higher risk of bronchospasm and laryngospasm.

Additionally, the use of volatile anesthetic agents increases the risk of operating room pollution.^8^

In our study, nearly half of the patients were in the 1-5 year age group, which is consistent to findings from other studies.^13^ Five patients were over 15 years old and underwent bronchoscopy for the removal of various foreign bodies. As seen in most other studies, a male predominance was observed.^13^ Additionally, the type and duration of foreign body have important anesthetic implications in such cases.^14-15^ Organic foreign bodies tend to swell over time, leading to airway obstruction and some cases may remain undiagnosed until secondary infections occur. If a patient presents with increased oxygen requirements, it indicates pre-existing lung compromise, which makes it more difficult to maintain adequate oxygenation during the procedure. In our study, the most frequently encountered foreign bodies were organic seeds (19 cases), including two repeat cases. Various age groups present with different types of foreign bodies. For example, pen back cover is common in the 5-15-year age group and may be difficult to remove due to their shape. One case in our study required a repeat bronchoscopy and ultimately tracheostomy for the removal of foreign body.

Foreign body retrieval was successful in 73% of cases, and the rate of negative bronchoscopy was 14.6%, which aligns with findings from a study conducted in India.^16^ Of the six negative bronchoscopy cases, two were followed up post-procedure. In one case, the patient who had excessive vomiting and drooling of saliva, underwent a rigid esophagoscopy the next day, which revealed a bean lodged in the esophagus. In another case, a hijab pin initially visible in the lung field on a chest X-ray was not located during bronchoscopy. Follow-ups X-ray later confirmed that the pin had migrated to the stomach. Twelve percent of the cases were referred for thoracotomy.

## CONCLUSION

Desaturation was the most common intraoperative complication. About half of the patient required mechanical ventilator postoperatively. Glycopyrrolate and steroids were used for preoperative preparations. Intravenous anesthesia was most commonly used for induction and maintenance. Almost two-third bronchoscopies achieved succesful foreign body removal.
